# Human enterovirus 71 subgenotype B3 lacks coxsackievirus A16-like neurovirulence in mice infection

**DOI:** 10.1186/1743-422X-2-74

**Published:** 2005-08-26

**Authors:** Yoke-Fun Chan, Sazaly AbuBakar

**Affiliations:** 1 Sime Darby Technology Centre, 2, Jalan Tandang, 46050 Petaling Jaya, Selangor, Malaysia; 2 Department of Medical Microbiology, Faculty of Medicine, University of Malaya, 50603 Kuala Lumpur, Malaysia

## Abstract

**Background:**

At least three different EV-71 subgenotypes were identified from an outbreak in Malaysia in 1998. The subgenotypes C2 and B4 were associated with the severe and fatal infections, whereas the B3 virus was associated with mild to subclinical infections. The B3 virus genome sequences had ≥85% similarity at the 3' end to CV-A16. This offers opportunities to examine if there are characteristic similarities and differences in virulence between CV-A16, EV-71 B3 and EV-71 B4 and to determine if the presence of the CV-A16-liked genes in EV-71 B3 would also confer the virus with a CV-A16-liked neurovirulence in mice model infection.

**Results:**

Analysis of human enterovirus 71 (EV-71) subgenotype B3 genome sequences revealed that the 3D RNA polymerase and domain Z of the 3'-untranslating region RNA secondary structure had high similarity to CV-A16. Intracerebral inoculation of one-day old mice with the virus resulted in 16% of the mice showing swollen hind limbs and significantly lower weight gain in comparison to EV-71 B4-infected mice. None of the mice presented with hind leg paralysis typical in all the CV-A16 infected mice. CV-A16 genome sequences were amplified from the CV-A16-infected mice brain but no amplification was obtained from all the EV-71-inoculated mice suggesting that no replication had taken place in the suckling mice brain.

**Conclusion:**

The findings presented here suggest that EV-71 B3 viruses had CV-A16-liked non-structural gene features at the 3'-end of the genome. Their presence could have affected virulence by affecting the mice general health but was insufficient to confer the EV-71 B3 virus a CV-A16-liked neurovirulence in mice model infection.

## Background

Enterovirus 71 (EV-71) was first described in 1969 during an outbreak with central nervous system complications in California [1]. Since then, EV-71 infections have been associated with a number of outbreaks with wide clinical manifestations, ranging from mild hand, foot and mouth disease (HFMD) to severe neurological complications and deaths. These include outbreaks in Bulgaria [2], Hungary [3], Japan [4] and more recently Malaysia [5,6], Taiwan [7] and Singapore [8]. In the later three outbreaks, more than a hundred deaths in total were reported, elevating EV-71 infection as one of the most deadly virus infection to date amongst young children below the age of 3 years old in Asia. The sudden emergence of the deadly forms of EV-71 infection in Asia was puzzling, as the virus together with other *human enterovirus A *viruses especially coxsackievirus A5 (CV-A5), CV-A10 and CV-A16 have been noted to cause HFMD in the region for sometime [9]. During the outbreak in Malaysia, at least three different EV-71 subgenotypes were identified. The subgenotypes C2 and B4 were associated with the severe and fatal infections, whereas, mild to subclinical infections were associated with the B3 viruses [10-12]. Unlike the earlier two subgenotypes, the B3 virus circulated for only a brief period during the outbreak and they have since not been isolated from patients from the later outbreaks [11,12]. A recent study reported that the B3 virus genome sequences had ≥93% similarity to EV-71 at the 5' end whereas the P3 genome region and 3'UTR had ≥85% similarity to CV-A16 [13]. CV-A16 is known to be the most common causative agent for the self-limiting HFMD. It is usually characterized by mild fever, oral ulcers and vesicular lesions on palms and soles and is not known to cause severe and fatal CNS infections. It is not presently understood why EV-71 infections tend to cause the more severe form of HFMD in comparison to CV-A16. The findings that EV-71 B3 viruses had high sequence similarity to CV-16 at the 3' end of the genome and that the viruses were not associated with the severe form of HFMD, offered opportunities to examine the potential roles of the respective genes in determining virulence. Hence, the present study was undertaken to examine if there are characteristic similarities and differences between CV-A16, EV-71 B3 and the more virulent EV-71 B4 virus and to determine if the presence of the CV-A16-liked genes in the EV-71 B3 virus genome would also confer the virus a CV-A16-liked neurovirulence in mice.

## Results and Discussion

**Figure 1:**
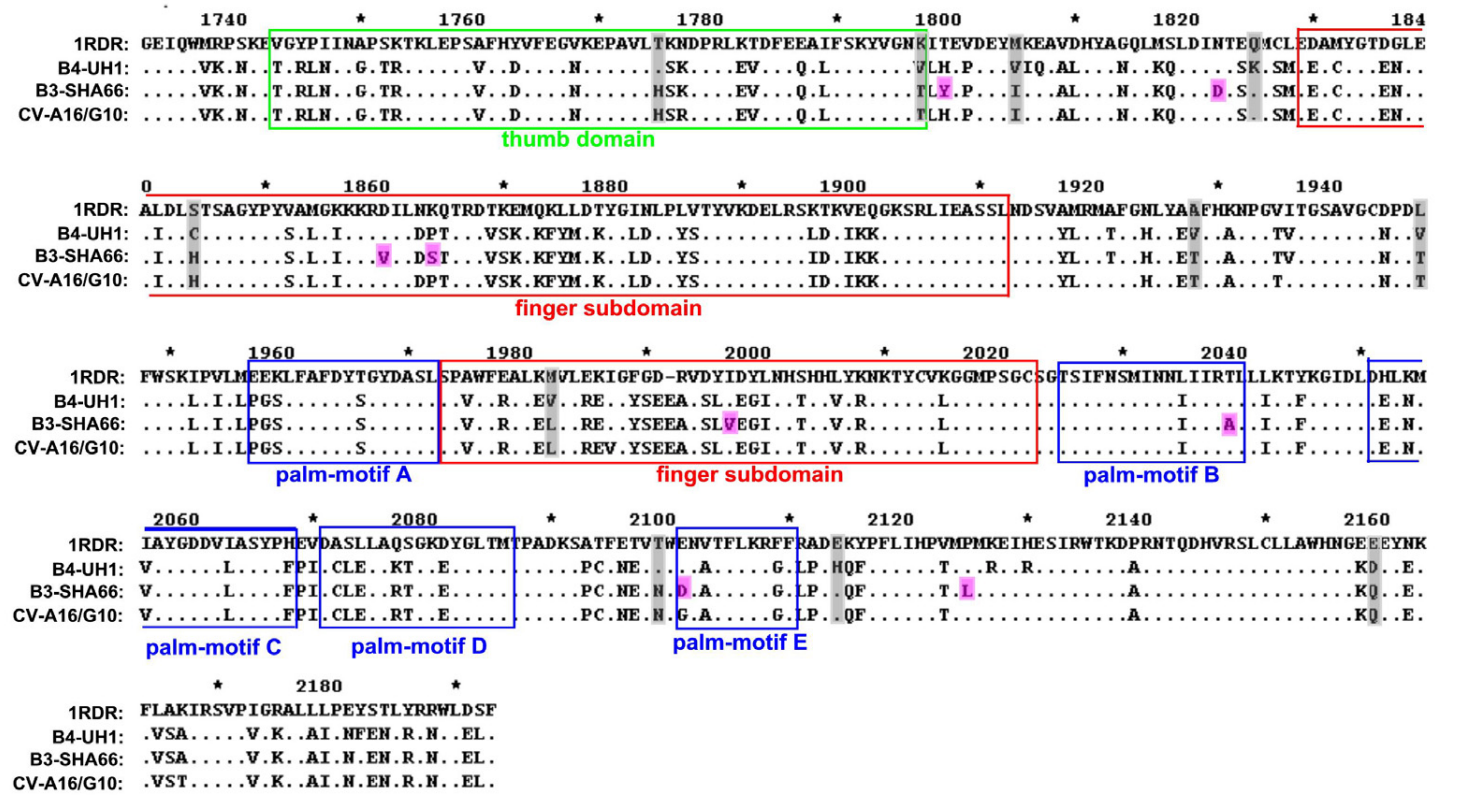
Structural alignment of EV-71 and CV-A16 3D RNA polymerase amino acid sequences. EV-71 subgenotype B3, B4 and CV-A16/G10 amino acid sequences were aligned against the poliovirus 1 Mahoney 3D RNA polymerase template sequences (PDB: 1RDR). Conserved residues are indicated as (●) and each domain are boxed and labeled. Residues shared by EV-71 B3 virus and CV-A16 were highlighted in grey and residues unique for EV-71 B3 virus were highlighted in pink.

**Figure 2:**
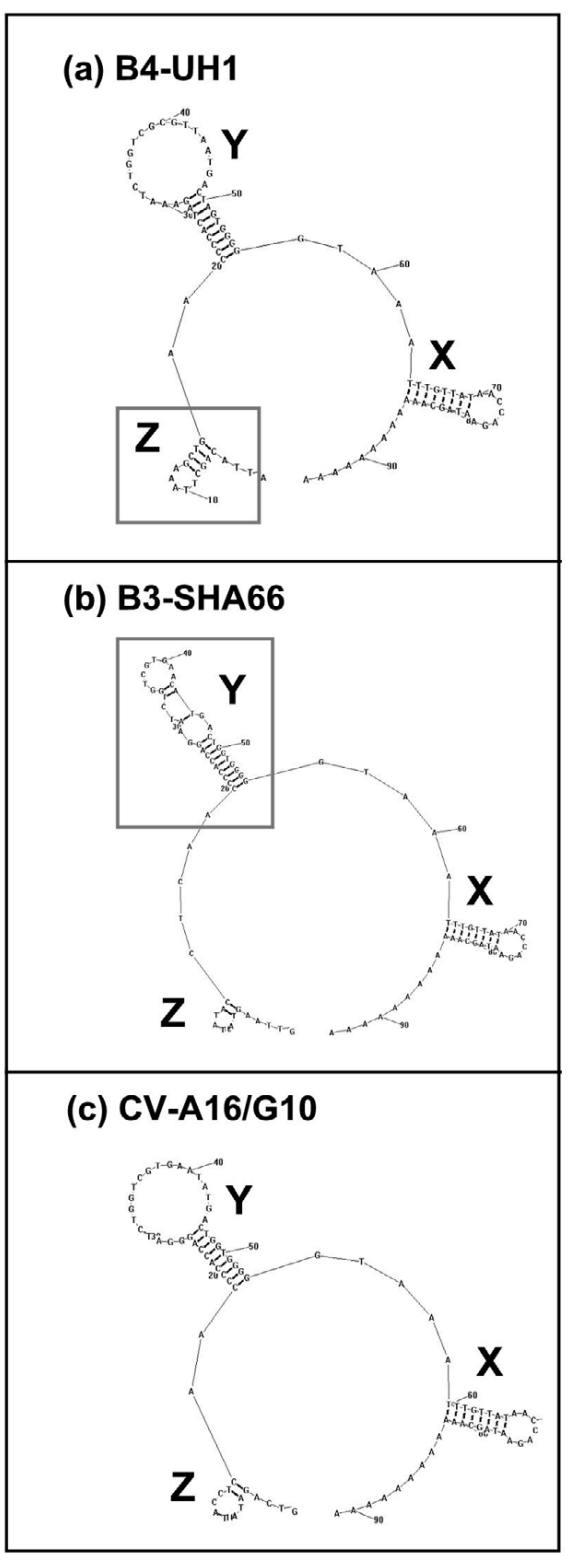
Predicted RNA secondary structures of EV-71 B3, EV-71 B4 and CV-A16/G10 3' UTR. RNA structures were predicted based on the lowest free energy, using the Zuker algorithm as implemented in RNA Structure (version 3.71). The predicted 3' UTR structures consisted of nucleotides from position 7326–7407 and additional 12 nucleotides of the poly-A tail.

The consensus amino acid sequences of the two available EV-71 B3 virus genomes (SHA63 and SHA66) were compared to other available subgenotype B4 and CV-A16/G10 genome sequences from the Genbank. Several amino acids (His^1775^, Thr^1947^, Ile^1806^, Gln^1825^, Thr^1928^, Thr^1947^, Asn^2099^, Glu^2114 ^and Gln^2159^) that were characteristic of the CV-A16/G10 were found in EV-71 B3 isolates. These amino acid differences occurred only within the 3D RNA polymerase gene, suggesting that this gene is very much CV-A16 than it is EV-71. Comparisons of the EV-71 B3 amino acid sequences against all other EV-71 and CV-A16 also revealed at least 12 amino acids (Asn^1124^, Arg^1152^, Ser^1335^, Ser^1641^, Tyr^1799^, Asp^1822^, Val^1860^, Ser^1864^, Val^1997^, Ala^2039 ^Asp^2101 ^and Leu^2125^) that were unique to the EV-71 B3 isolates. Eight of these amino acid differences occurred within the 3D RNA polymerase gene. Two of these unique mutations found were located between amino acids 176–348 genome region essential for RNA-protein interactions [14] (Fig. 1). Alignment of the EV-71 B3 (SHA66) and EV-71 B4 (UH1) isolates RNA polymerase against the three-dimensional crystal structure of poliovirus 1 Mahoney strain 3D RNA polymerase (PDB: 1RDR) was performed to locate these mutations. Of these eight mutations in EV-71 B3 virus, three were located within the finger subdomain and two were located at the palm motif suggesting that the EV-71 B3 virus amino acid substitutions were mainly located within the 3D RNA polymerase functional domains. The highly 'flexible' finger domain is involves in modulating substrate recognition and oligomerization of the polymerase for binding to nucleotides [15]. In poliovirus, mutations within the 3D RNA polymerase located to the 3' end of the genome have been shown to affect neurovirulence [16,17]. Hence, this highlights the potential importance of the 3D RNA polymerase in determining the virus neurovirulence. It was also found that in addition to the presence of CV-A16 or CV-A16-liked 3D RNA polymerase gene sequences, the EV-71 B3 viruses also shared a similar predicted 3' UTR secondary structures with CV-A16/G10 at domain Z (Fig. 2), a domain reported as important in determining cardiovirulence of CV-B3 [18]. Mutations that affect the stem-and-loop structures have been shown earlier to abolish infectivity and virus RNA synthesis [19,20]. The predicted domain Y known to form a tertiary RNA 'kissing' structure with domain X of the EV-71 B3 virus, however, differed from the EV-71 B4 and CV-A16/G10 (Fig. 2).

**Figure 3:**
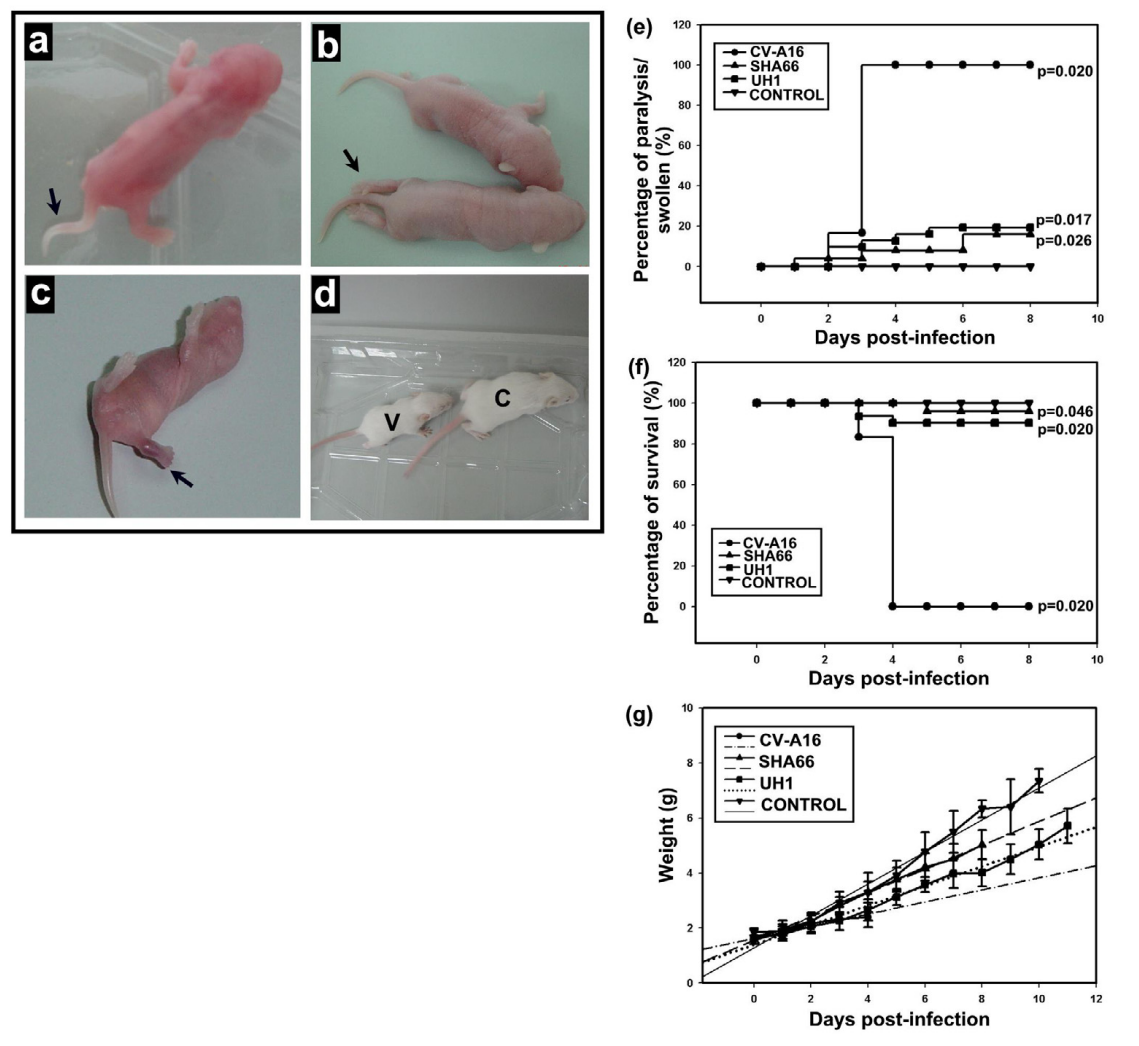
EV-71 and CV-A16 infections of newborn mice. One-day old newborn mice were intracerebrally inoculated with 1 × 10^3 ^PFU virus per mouse and monitored daily. CV-A16-infected mice had floppy tails on day two post-inoculation (a) and hind leg paralysis beginning on day three post-inoculation (arrow, b). Mice with swollen limbs were noted in EV-71 B3 virus infection (arrow, c) and the EV-71 B3-infected mice had significantly reduced body weight gain in comparison to the mock-infected mice (d, V = B3-infected mouse, C = mock-infected mouse). Mice with floppy tails, swollen limbs and paralysis (e) and death (f) were recorded. The weight gain of the surviving mice was also determined (g).

**Figure 4:**
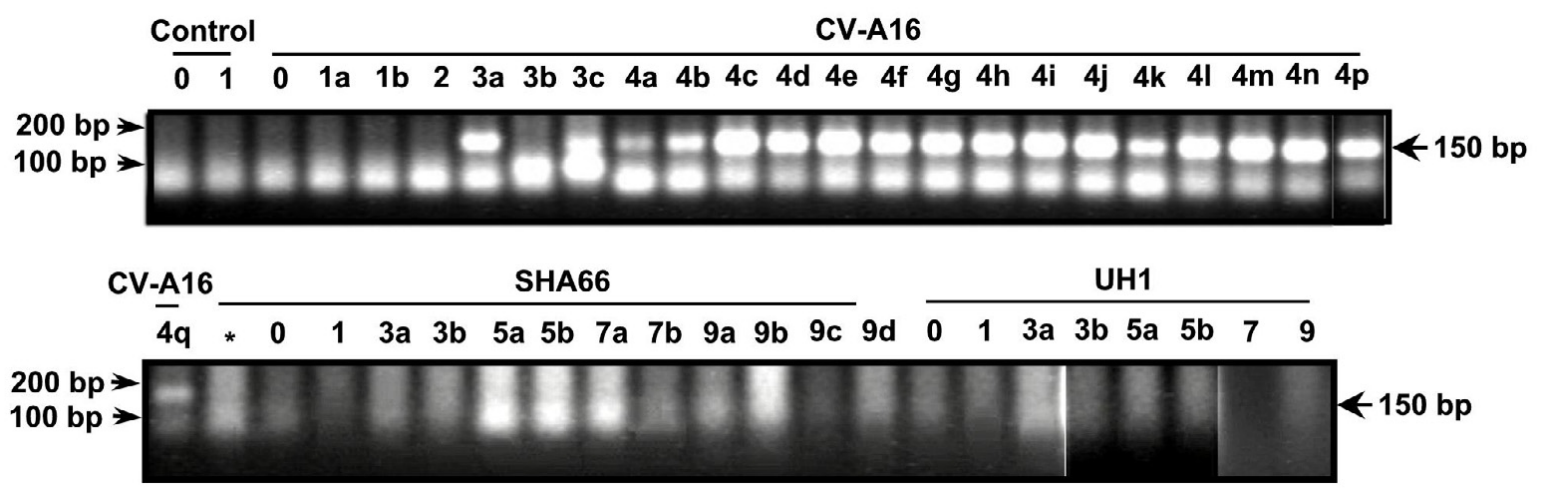
Detection of enterovirus genome sequences in infected newborn mice brain. At selected intervals post-inoculation (indicated by the number above each lane), mice were sacrificed (each mouse indicated by the alphabet above each lane) and RT-PCR was performed using an enterovirus specific primers. The presence of a 150 bp amplified DNA fragment indicates the presence of enterovirus genome, which was later confirmed by DNA sequencing.

Inoculation of one day-old newborn mice showed that all mice inoculated with CV-A16 had the typical signs and symptoms of CV-A16 infections by day two post-inoculation. The mice were lethargic, had floppy tails, tremoring, uncoordinated movement and reduced average body weight in comparison to EV-71 B3- or EV-71 B4-inoculated mice (Fig. 3a,3g). Approximately 17% (4/24) of the mice had hind leg paralysis by day three post-inoculation and one died (Fig. 3b,3e,3f, Additional file: 1). By day four post-inoculation, all the CVA16-inoculated mice had developed hind leg paralysis and subsequently died (Fig. 3b,3e,3f). A 150 bp enterovirus genome sequence were amplified and sequenced from the total RNA of the brain of all the CV-A16-inoculated mice confirming the presence of CV-A16 in the mice brain (Fig. 4). Mice-inoculated with EV-71 B3 and EV-71 B4 viruses also had significantly reduced average body weight in comparison to the control mock-infected mice (Student's *t*-test, *P *< 0.05, Fig. 3d,3g). Mice inoculated with EV-71 B3 virus, however, had significantly reduced average body weight in comparison to those inoculated with the EV-71 B4 virus (Fig. 3g). These mice appeared lethargic and uncoordinated beginning on day two post-inoculation. Of these, 16% (4/25) developed swollen hind legs and one subsequently died on day five post-inoculation (Fig. 3c,3e,3f). There were no hind leg paralysis noted and the remaining surviving mice recovered, fed well and regained balance after day six post-inoculation. In contrast, about 20% (6/31) of the mice inoculated with EV-71 B4 virus developed swollen fore limbs or hind legs and of these, three died after day four post-inoculation (Fig. 3e,3f). After day eight post-inoculation, the B4-inoculated mice also recovered, became more active and fed well. Pairwise comparison of the clinical illness and survival probability between the virus-inoculated groups and control were significant suggesting that the three viruses, CV-A16, EV-71 B3 and EV-71 B4 viruses caused death in mice (log rank survival analysis, *P *< 0.05, Fig. 3e,3f) but only infection with CV-A16 lead to 100% mortality. In contrast to CV-A16 infection, no amplification of the enterovirus sequence was detected in the selected EV-71 B4- and EV-71 B3-inoculated mice brain, suggesting that EV-71 B3 and EV-71 B4 viruses perhaps did not replicate in the mice brain when introduced intracerebrally (Fig. 4). This may help to explain the absence of hind leg paralysis in all the EV-71-infected mice and the complete recovery of all the surviving mice. Death seen amongst these mice may have been caused by infection of other tissues as manifested in mice with swollen limbs and legs. Evidence suggesting that EV-71 strains isolated during the Bulgaria poliomyelitis-like epidemic had higher tropism for mouse muscle tissues than the brain tissues [2] and EV-71 neurovirulence mimicking human infection was achieved only from using a mouse-adapted virus strain but not the parental strain [21,22] support the findings from the present study that EV-71 B3 and B4 did not infect the brain. The infection, however, manifests clinically in some mice as non-specific swollen limbs and legs. Hence it is possible that, though both EV-71 and CV-A16 viruses are closely related, different receptors are utilize for the respective virus entry into the different tissues and this could be mediated through the virus structural proteins. The mutations that occurred within the 3D RNA polymerase of the EV-71 B3 virus along with the presence of CV-A16-liked 3' UTR domain Z RNA secondary structure then could contribute to virulence but by themselves did not affect EV71 neurovirulence in mice as in contrast to CV-A16, the B3 virus lacks tropism for the mice brain. Since the major differences between the EV-71 B4 and EV-71 B3 viruses occurred at the 3' end of the genome, this support the view that the structural genes of EV-71 and CV-A16 determined tissue tropisms.

Results from the present study, also did not support the possibility that acquisition of CV-A16-liked genome sequences alone is sufficient to confer the EV-71 B3 virus a CVA16-liked neurovirulence in mice. The significant mice weight gain differences noted between mice infected with EV-71 B3 and EV-71 B4 viruses, with the later performing much better, however, suggested that EV-71 B3 virus infection somehow did affect mice general health. As weight gain differences are the only biological parameter that differentiate between the B3 and B4 viruses, it does appears that EV-71 B3 affected mice more than the EV-71 B4 virus. It is also worth noting that in contrast to infection in mice, CV-A16 infection in human in general does not result in severe infection as oppose to EV-71, particularly the EV-71 B4 virus infection. In parallel manner, the EV-71 B3 viruses, while they affected mice, they did not cause severe or fatal infection in humans. These implied that the EV-71 B3 virus is truly different and as its genome suggested, it has to some extent features of both EV-71 and CV-A16 infection *in-vivo*.

## Conclusion

Results from the present study suggest that EV-71 B3 virus had CV-A16-liked non-structural gene 3D RNA polymerase and 3' UTR features at the 3' end of the genome. Their presence affected virulence differently from infection with EV-71 B4 and CV-A16 by affecting the mice general health. The presence of the CV-A16-liked genes, however, was insufficient to markedly influence the neurovirulence properties of EV-71 B3 virus in mice.

## Materials and methods

### Viruses

Two EV-71 isolates identified from the 1997 HFMD outbreak in Malaysia were used. The subgenotype B3 isolate, SHA66 (EMBL: AJ238457) was isolated from a HFMD patient presented with mild infection [6,23]. The subgenotype B4 isolate, UH1 (EMBL: AJ238455) on the other hand, was isolated from the brain of a patient who died of EV-71-associated neurogenic pulmonary edema [5,6,24]. The CV-A16 isolate used was previously isolated from a HFMD patient seen at the University Malaya Medical Centre. This CV-A16 isolate was identified and characterized using monoclonal antibody staining (Chemicon Cat #3323, California, USA) and amplification of partial 5' UTR gene (data not shown).

### Amino acid sequence analysis

Amino acid sequences were examined after stripping the 5' UTR and 3' UTR sequences and consensus sequences of EV-71 B3 and EV-71 B4 viruses were aligned and manually edited using GeneDoc software [25]. The previously published three-dimensional crystal structure of the 3D RNA polymerase was downloaded as template for the alignment. Using the WHAT IF program [26], domains that represent the conserved regions, loops, insertion or deletions were manually visualized to generate a structural alignment.

### RNA secondary structure prediction

The 3' UTR RNA secondary structure was predicted using Zuker optimal and suboptimal minimal free energy folding algorithms, as implemented in RNA Structure version 3.71 software [27]. Part of the poly A tract was incorporated into the sequences.

### Determination of virulence in mice

A total of 24, 25 and 31 one-day old newborn ICR mice were inoculated intracerebrally with either CV-A16 or SHA66 (B3 virus) or UH1 (B4 virus) virus inoculum. The virus inoculum with infectivity of ~1 × 103 p.f.u. was injected in a volume of 10–20 μl into the mice brain. The mice were closely monitored for any clinical symptoms, paralysis and death and the weight of each surviving mouse was recorded daily up to day 11 post-inoculation. Another litter with at least 10 one-day old newborn mice was injected with comparable growth medium and used as controls. At selected intervals post-infection, some of the mice were sacrificed and the brain tissues were harvested for total RNA using the TRI Reagent™ (Molecular Research Centre, Inc., Cincinnati, USA) following the manufacturer's recommended protocols. The RT-PCR amplification for the detection of enterovirus sequence was performed using 1 μg of RNA. Access RT-PCR kit (Promega, USA) and primer pairs, EntabF (5'-TCC TCC GGC CCC TGA ATG CGG CTA AT-3'; nucleotide positions 449–474, based on MS87 strain, Genbank: U22522) and EVRR (5'-AAT TGT CAC CAT AAG CAG GC-3'; nucleotide positions 586–606) were used. Reverse transcription was performed at 42°C for an hour followed by amplification steps; 95°C-30 seconds, 55°C-30 seconds and 72°C-30 seconds for 30 cycles and finally with 5 minutes extension at 72°C using the PTC thermal cycler (MJ Research, Massachusetts, USA). When no amplicon was obtained, the number of cycle was increased to 40. Alternatively, a second step PCR using similar parameters was performed using ten-fold diluted RT-PCR product as template. The amplified DNA fragments were electrophoresed using 2% agarose gel in 0.5 × tris-acetate EDTA buffer (0.02 M Tris base, 0.5 mM EDTA pH 8.0, 0.057% glacial acetic acid) and sequence confirmation was made by sequencing the DNA fragment.

### Statistics

Student's *t*-test was used to evaluate if the differences in weight between the virus-inoculated mice and control mice was significant. Wilcoxon signed rank test was used to compare the survival and paralysis probability between the virus-inoculated mice and control mice. All statistical analyses were implemented using SPSS for Windows version 11.5 (SPSS Inc., Illinois, USA). All tests were two-sided and *P *< 0.05 was considered as statistically significant.

## List of Abbreviation

CV Coxsackievirus

EV Enterovirus

HFMD Hand, foot and mouth disease

UTR Untranslated region

## Competing interests

The author(s) declare that they have no competing interests.

## Authors' contributions

The corresponding author, Sazaly AbuBakar is the principal investigator of the study; is involved in the design, supervision, data analyses and writing of the report. Chan Y-F performed all the virological investigations, nucleotide sequencing and analyses of data. All authors were involved in the preparation of this "*Research Article*" and figures.

## Supplementary Material

Additional File 1 Hind leg paralysis in CV-A16 infected mice. By day three post-inoculation, the mice were lethargic, tremoring and uncoordinated. Click here for file
